# Modulating autonomic nervous system activity with transcutaneous auricular vagus nerve stimulation in Parkinson’s disease: a proof of concept study

**DOI:** 10.3389/fnins.2026.1830260

**Published:** 2026-07-02

**Authors:** Alexandra Evancho, W. J. Tyler

**Affiliations:** 1 Center for Engagement in Disability Health and Rehabilitation Science (CEDHARS), Department of Physical Therapy, The University of Alabama at Birmingham, Birmingham, AL, United States; 2 Department of Biomedical Engineering, The University of Alabama at Birmingham, Birmingham, AL, United States

**Keywords:** autonomic nervous system dysfunction, dysautonomia, Parkinson’s disease, transauricular vagus nerve stimulation, vagus nerve stimulation

## Abstract

**Background:**

Cardiovascular dysautonomia is a debilitating non-motor symptom of Parkinson’s disease (PD) that limits exercise capacity and neurorehabilitation outcomes. Transcutaneous auricular vagus nerve stimulation (taVNS) is an emerging non-invasive neuromodulatory therapy that modulates cardiovascular activity and could potentially serve as an adjunct to exercise, yet its physiological effects on cardiovascular function in PD remains unexplored.

**Objective:**

This proof-of-concept, sham-controlled crossover pilot study (*N* = 8) investigated the acute effects of taVNS on cardiovascular autonomic activity in idiopathic PD.

**Methods:**

Participants underwent active taVNS (30 Hz, 250 μs, 0.1–4 mA) or sham stimulation (0 mA) during a 15-min resting phase, immediately followed by the Ewing Battery of cardiovascular reflexes. Acute autonomic shifts were phenotyped using continuous heart rate variability (HRV) monitoring.

**Results:**

This proof-of-concept protocol was feasible, as all participants completed the randomized crossover stimulation visits and autonomic reflex testing without adverse events. Baseline autonomic burden (COMPASS-31) was associated with the magnitude of heart rate response to active stimulation. Immediately following stimulation and during the deep breathing challenge, active taVNS was associated with directionally consistent changes in vagally mediated HRV metrics including RMSSD, pNN50, and HF power, relative to sham.

**Conclusion:**

Continuous HRV monitoring and autonomic reflex testing appears feasible for characterizing acute autonomic responses to taVNS in PD. Active stimulation was associated with directional changes in vagally mediated HRV metrics during the post-stimulation period and during deep breathing, supporting the biological plausibility of acute autonomic modulation. These preliminary findings justify larger, adequately powered studies designed to determine whether taVNS can reliably modulate cardiovascular autonomic regulation and inform rehabilitation optimization in PD.

## 1 Introduction

Parkinson’s Disease (PD) is a degenerative neurologic condition characterized by both motor and non-motor symptoms (Rissardo et al., 2025; Chen et al., 2020; Pfeiffer, 2020). Although clinical attention is typically centered on motor symptomology, non-motor symptoms are pervasive and substantially impact quality of life (Rissardo et al., 2025). Among these, cardiovascular (CV) dysautonomia is a non-motor PD symptom arising from degeneration of central and peripheral autonomic pathways that regulate heart rate (HR) and blood pressure (BP; Cuenca-Bermejo et al., 2021). Clinically, CV dysautonomia can manifest as orthostatic hypotension (OH), chronotropic incompetence (CI), and supine hypertension (Chen et al., 2020; Palma and Kaufmann, 2020; Kim et al., 2016; Griffith et al., 2024). CV dysautonomia is associated with increased mortality and falls risk (Maule et al., 2012; Kamo et al., 2026), and may also interfere with a patient’s ability to effectively engage in exercise (Sabino-Carvalho and Vianna, 2020).

Exercise and physical therapy are well-established non-pharmacologic interventions that can improve both motor and non-motor PD symptoms (Mak et al., 2017; Li et al., 2021; Johansson et al., 2020; Hirsch et al., 2018; Gilat et al., 2021; Feng et al., 2020; Ernst et al., 2023). Achieving moderate-to-high exercise intensity is particularly important for optimizing therapeutic benefit (Schenkman et al., 2018; Ramos et al., 2024; Kathia et al., 1985). However, this level of exertion requires an appropriately coordinated CV response to meet increasing metabolic demands of working skeletal muscle. CV dysautonomia in PD is multifaceted, reflecting the widespread impact of PD pathology on both sympathetic (SNS) and parasympathetic (PNS) branches of the autonomic nervous system (ANS; Goldstein et al., 2000; Ziemssen and Reichmann, 2010). Under normal conditions, the SNS and PNS work synergistically to regulate heart HR and BP during physical activity, in part through the baroreflex reflex arc, which maintains BP stability via dynamic adjustments in cardiac output and vascular tone (White and Raven, 2014; Benarroch, 2008). In PD, impaired baroreflex sensitivity and cardiac sympathetic denervation contribute to a blunted or abnormal CV response to exercise (Griffith et al., 2024; Sabino-Carvalho and Vianna, 2020; Kanegusuku et al., 2022; Roberson et al., 2019; Sabino-Carvalho et al., 2018; Miyasato et al., 2018). Accordingly, interventions that target and improve autonomic regulation, including baroreflex function, may represent a novel strategy to address CV limitations, improve CV response to exercise, and enhance rehabilitation outcomes in this population.

Transcutaneous auricular vagus nerve stimulation (taVNS) is a promising, non-invasive neuromodulatory intervention capable of safely influencing CV and baroreflex activity (Yoshida et al., 2025; Ackland et al., 2025; Hatik et al., 2023; Yokota et al., 2022; Forte et al., 2022; Carandina et al., 2021; Clancy et al., 2014; Gentile et al., 2025). taVNS delivers low-intensity electrical currents to the auricular branch of the vagus nerve (ABVN), increasing afferent input to the nucleus tractus solitarius (NTS; Martin et al., 2024; Keute and Gharabaghi, 2021; Collins et al., 2021; Urbin et al., 2021; Yap et al., 2020). The NTS, located in the brainstem, serves as the primary integration center for CV autonomic function (Machado et al., 1997). By increasing afferent input to the NTS, taVNS may modulate downstream autonomic output and improve coordination of HR and BP regulation. In healthy adults, taVNS has been shown to improve baroreflex sensitivity (Antonino et al., 2017) and improve measures of cardiorespiratory fitness and increase exercise capacity (Ackland et al., 2025), supporting the hypothesis that taVNS could be leveraged to improve CV responses to exercise in PD. However, the effects of taVNS on CV autonomic function in this population remain poorly understood.

Therefore, the primary purpose of this proof-of-concept study was to establish a framework for assessing CV autonomic responses to taVNS in individuals with PD. To achieve this, we evaluated autonomic function at rest and during standardized autonomic challenges using the Ewing Battery, a clinical set of autonomic reflex tests including orthostatic assessment, deep breathing, and the Valsalva maneuver (Ewing et al., 1985; Ewing and Clarke, 1982). This approach was designed to characterize how taVNS influences CV regulation across different physiological conditions, including responses mediated by the baroreflex pathways. Secondary, exploratory aims were to characterize preliminary patterns in these responses, including changes in heart rate variability (HRV) as an index of autonomic balance, and to examine whether baseline autonomic symptom burden influenced physiological responsiveness to taVNS. Collectively, these analyses were intended to provide early insight into potential responder profiles and to inform future studies aimed at strategically leveraging autonomic modulation to enhance rehabilitation outcomes in patients with PD.

## 2 Methods

### 2.1 Study design and participants

**Figure 1:**
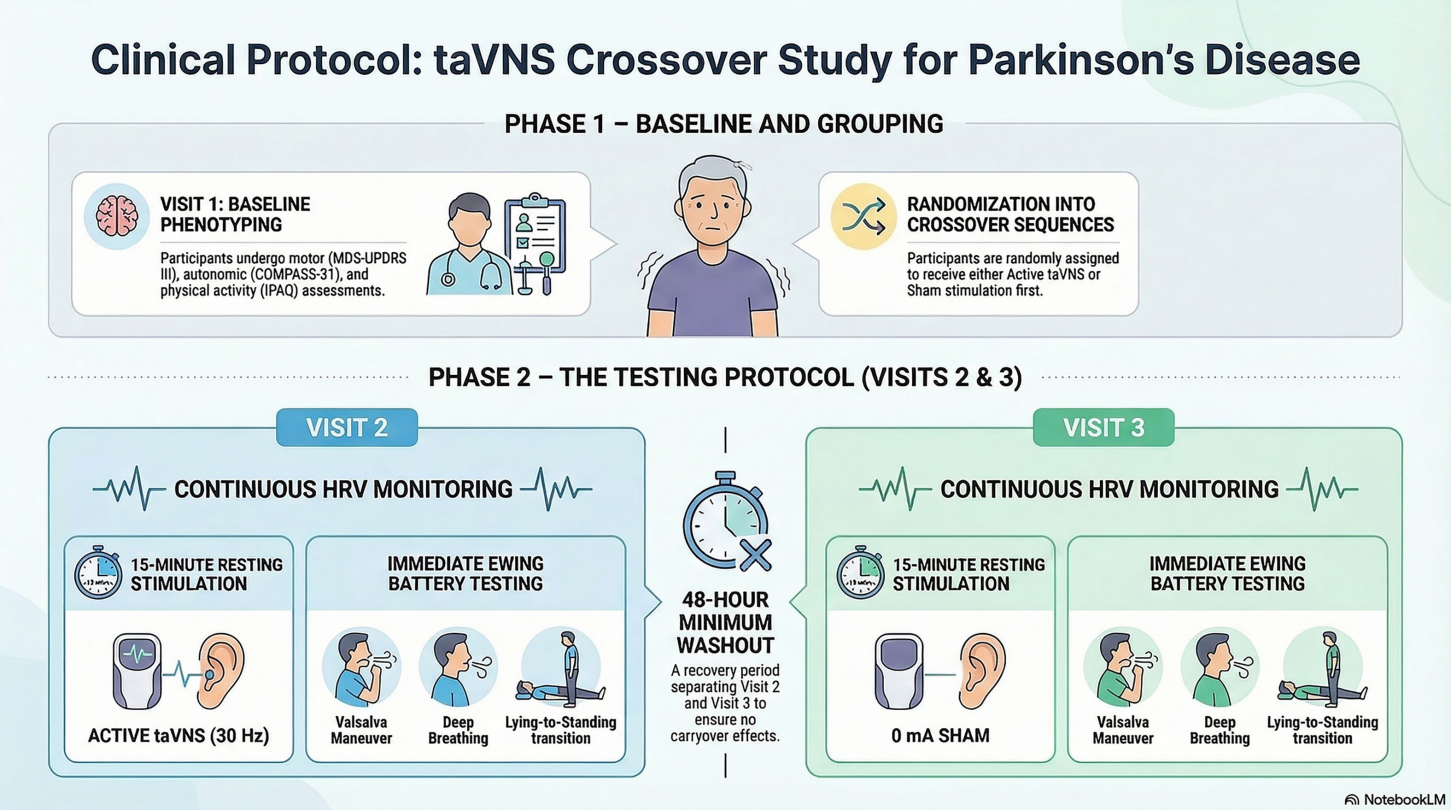
Randomized, sham-controlled, crossover study design and intra-visit protocol. Flowchart detailing the experimental design for evaluating the acute autonomic effects of transcutaneous auricular Vagus Nerve Stimulation (taVNS) in an idiopathic Parkinson’s disease cohort (N = 8). Following baseline phenotyping, participants were randomized into one of two crossover sequences, receiving either active taVNS or sham stimulation during their first interventional visit, and crossing over to the alternate condition during the subsequent visit. A minimum 48-h washout period was observed between visits. The lower panel illustrates the standardized intra-visit testing timeline. Continuous autonomic monitoring (electrocardiography and hemodynamics) was maintained throughout four sequential epochs: a 1-min pre-treatment baseline, a 15-min stimulation phase, a 1-min post-treatment washout, and the dynamic Ewing autonomic test battery (comprising the Valsalva maneuver, deep breathing, and a lying-to-standing orthostatic challenge). Active taVNS was delivered at 30 Hz with a 250 μs pulse width, titrated to each individual participant’s sub-perceptual sensory threshold; sham stimulation was delivered at 0 mA.

A randomized, single-blind, sham-controlled, within-subjects crossover pilot study was conducted to evaluate the acute effects of taVNS on CV autonomic function in *N* = 8 individuals with idiopathic PD. Eligible participants were aged 35 to 80 years, independently ambulatory, and maintained on a stable dopaminergic medication regimen for at least 4 weeks prior to the study. Exclusion criteria strictly prohibited the participation of individuals with the use of beta-blockers, sustained, severe hypertension (180/110 mmHg while seated or supine), significant uncontrolled cardiac arrhythmia, unstable angina, congestive heart failure, a history of myocardial infarction, a history of seizures, and severe cognitive impairment. To prevent physiological carryover effects between the active and sham stimulations, a strict minimum 48-h washout period was mandated between the two testing visits. The study was approved by the Institutional Review Board at the University of Alabama at Birmingham (Protocol IRB-300014518) and adhered to the Declaration of Helsinki. All participants provided written informed consent. The study was registered on Clinicaltrials.gov (NCT07557706; Figure 1).

### 2.2 Clinical and behavioral phenotyping

To accurately characterize the cohort and establish baseline traits, participants underwent clinical and behavioral phenotyping prior to the intervention. Disease severity and motor impairment were assessed using the Movement Disorder Society Unified Parkinson’s Disease Rating Scale (MDS-UPDRS) Part III. Baseline autonomic symptom burden was quantified via the Composite Autonomic Symptom Score (COMPASS-31), and baseline exercise habits were evaluated using the International Physical Activity Questionnaire (IPAQ).

### 2.3 The taVNS intervention

Bilateral taVNS was delivered using a small, current-controlled device (vagus.net) connected to proprietary biocompatible, conductive polyvinyl alcohol (PVA) hydrogel earbud electrodes (BRAIN Buds; IST, LLC, Dover, DE, USA) inserted into the external acoustic meatus. At each visit, participants received 15 min of either active taVNS or a sham condition during a resting phase. For the active condition, the device delivered biphasic square wave pulses at a frequency of 30 Hz and a pulse width of 250 μs, with the current amplitude individualized to emit a current of 0.1 mA below each participant’s sensory threshold (ranging from 0.1 to 4.0 mA). During the sham condition, identical earbud electrodes were placed, but the device remained inactive (0 mA current output). Stimulation below perceptual threshold was selected to preserve participant blinding, as alternative sham approaches (e.g., stimulation of the earlobe) may also evoke measurable autonomic responses (Gharabaghi and Keute, 2025).

### 2.4 Autonomic stress testing (Ewing battery)

Immediately following the 15-min resting stimulation phase, participants underwent standardized CV reflex testing using the Ewing Battery (Ewing et al., 1985; Ewing and Clarke, 1982). These tests systematically perturb the ANS to measure its capacity to mount appropriate CV responses under dynamic physiological stress. The protocol included: (1) the Valsalva Maneuver, requiring participants to exhale forcefully into a mouthpiece to maintain a pressure of 40 mmHg for 15 s; (2) a Deep Breathing challenge, involving paced respiration at a controlled rate of 6 breaths per minute; and (3) an Orthostatic Challenge, where participants transitioned from a supine resting position to unassisted active standing.

### 2.5 Data acquisition and HRV analysis

CV hemodynamics were continuously monitored throughout the protocol using a Polar heart rate sensor. Raw interbeat interval (R-R) data were extracted and processed using Kubios HRV Scientific software to dynamically quantify sympathovagal shifts. To evaluate the temporal effects of the intervention, the continuous HRV data were segmented into three distinct epochs: a 1-min pre-treatment baseline, the 15-min treatment phase (active or sham), and a 1-min immediate post-treatment washout window. Subsequently, data acquired during the Ewing Battery were isolated into test-specific windows corresponding to each physiological challenge. Within these defined epochs, standardized HRV metrics were calculated in both the time domain (RMSSD, SDNN, and pNN50) and frequency domain (absolute LF power, HF power, and the LF/HF ratio). Furthermore, standard clinical autonomic reflex indices including the Valsalva ratio, deep breathing heart rate response, and the orthostatic 30:15 ratio were derived directly from the raw data utilizing the Kubios ANS Function Report module.

### 2.6 Statistical analysis

All statistical analyses were performed using the R statistical computing environment. Due to the sample size of this proof-of-concept pilot (*N* = 8) and the non-normal distribution of the data, non-parametric analytical approaches were utilized. Because of the within-subjects design, paired comparisons between the active taVNS and sham conditions (for both continuous HRV metrics and Ewing reflex responses) were conducted using the Wilcoxon Signed-Rank test. To establish predictive “responder profiles,” Spearman’s rank correlation coefficient (*ρ*) was utilized to identify relationships between baseline clinical phenotypes (COMPASS-31) and acute autonomic physiological deltas (the change in HRV metrics during active stimulation). Missing data were managed using pairwise deletion for each specific statistical comparison. Statistical significance was defined *a priori* as *p* < 0.05. Given the exploratory nature of this pilot study, all *p*-values should be interpreted as nominal. Analyses were therefore primarily descriptive, with emphasis placed on the direction, magnitude, and consistency of observed changes across study phases. Values that did not meet the conventional *p* < 0.05 threshold are reported only to aid interpretation of potential physiological patterns and to inform hypothesis generation for future studies; they were not interpreted as statistically significant or as evidence of efficacy.

## 3 Results

### 3.1 Cohort characteristics

A total of eight participants (*N* = 8) diagnosed with idiopathic PD completed the crossover pilot study. The cohort was predominantly male (63%) and white (88%), with a median age of 65.5 years (IQR: 59.5–71.0). Disease severity was characterized by a median MDS-UPDRS Part III motor score of 33 (IQR: 24–35), with the majority of participants (75%) at Hoehn and Yahr Stage 2. The median Levodopa Equivalent Daily Dose (LED) was 400 mg/day (IQR: 140–463). See Table 1 for baseline demographics. Paired comparisons did not identify significant between-condition differences in active and sham stimulation conditions regarding the time since the last medication dose (*p* = 0.147), the time since the last meal (*p* = 0.179), or the total caffeine consumed (*p* = 0.059). However, given the small sample size, the lack of statistically significant difference between groups does definitively not confirm that exogenous factors did not contribute to autonomic variability across visits.

**Table 1:** Baseline characteristic.

Baseline characteristic	*N* = 8^1^
Age (years)	65.5 [59.5–71.0]
Gender	
Male	5 (63%)
Female	3 (38%)
Race	
White	7 (88%)
Black	1 (13%)
Handedness	
Right	6 (75%)
Left	2 (25%)
Levodopa equivalent dose (mg/day)	400 [140–463]
COMPASS-31 total score	27 [19–32]
IPAQ (MET-minutes/week)	3,337 [1,752–9,382]
MDS-UPDRS Part III (Motor)	33 [24–35]
Hoehn and Yahr stage	
1	1 (13%)
2	6 (75%)
3	1 (13%)

1 Median [Q1–Q3]; *n* (%).

### 3.2 Feasibility, safety, and tolerability

The study protocol was feasible and well tolerated. All eight enrolled participants completed both study visits, including the active and sham stimulation conditions, continuous HRV monitoring, and the full Ewing Battery of autonomic reflex tests. No participants withdrew from the study, and no adverse events occurred during or immediately following either stimulation condition or during autonomic reflex testing. These findings support the feasibility and short-term safety of combining taVNS with continuous autonomic monitoring and standardized cardiovascular reflex testing in individuals with PD.

### 3.3 Responder profiling (baseline predictors)

**Figure 2:**
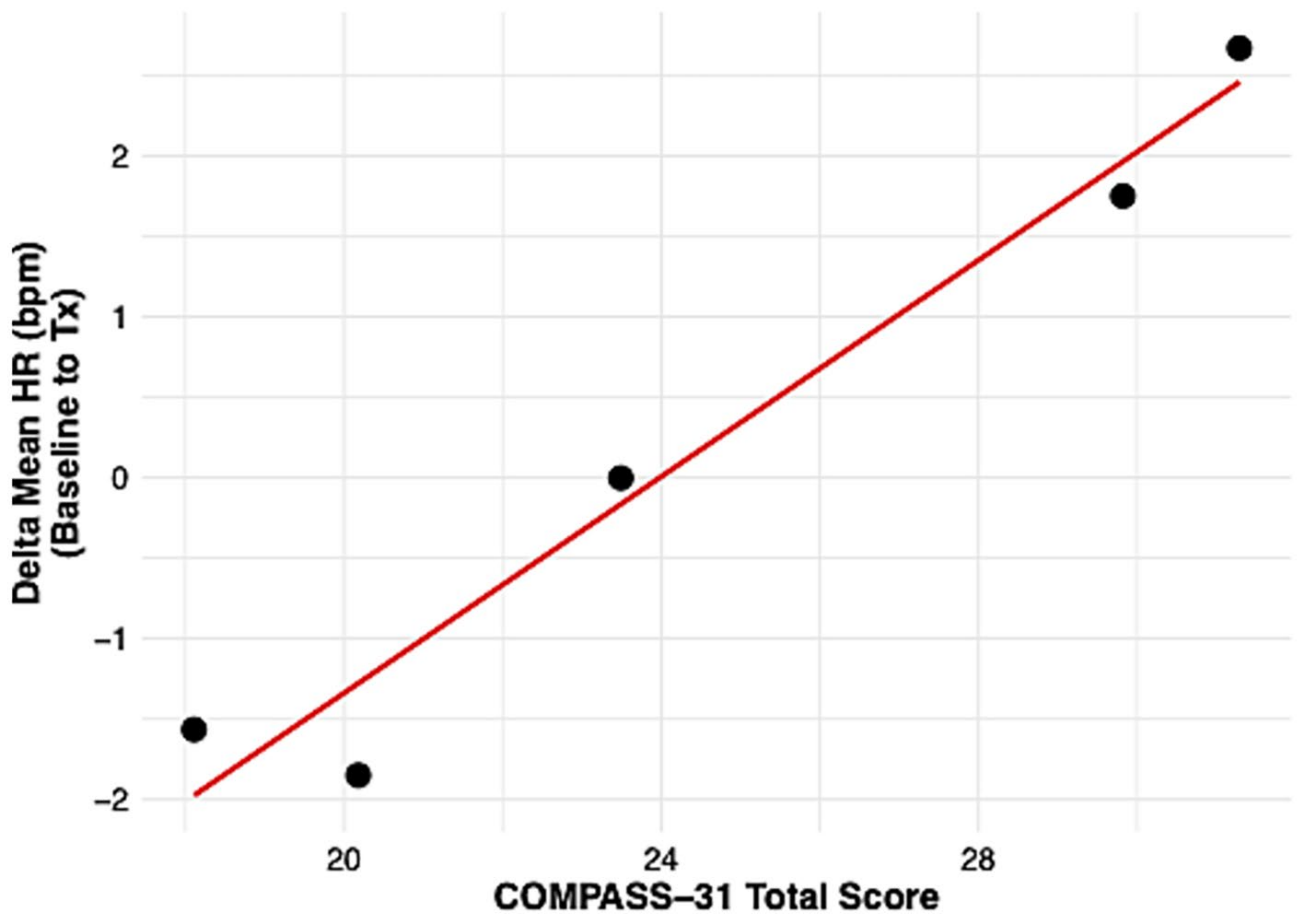
Responder profiles: Baseline autonomic symptom burden predicts acute chronotropic response to taVNS. Scatter plot demonstrating the relationship between baseline autonomic symptom severity and the magnitude of physiological response to active transcutaneous auricular Vagus Nerve Stimulation (taVNS) in individuals with Parkinson’s disease (*N* = 5). The x-axis represents the baseline Composite Autonomic Symptom Score (COMPASS-31) total score. The y-axis displays the change (delta) in mean heart rate (bpm) from the 1-min pre-treatment baseline epoch to the 15-min active stimulation phase. Individual patient values are represented by black circles, and the solid red line indicates the linear line of best fit. Statistical analysis utilized Spearman’s rank correlation, which suggests that individuals with a higher baseline autonomic burden exhibited a greater chronotropic response to active taVNS.

To explore potential physiological responder profiles, we evaluated the relationship between baseline clinical phenotypes and the acute physiological response to taVNS treatment (Δ values). Spearman’s rank correlations revealed an association between the participant’s baseline autonomic burden (measured by the COMPASS-31) and their heart rate response to active stimulation (*ρ* = 0.90, *p* = 0.037, Figure 2). Specifically, participants with greater baseline autonomic dysfunction (higher COMPASS-31 scores) exhibited larger changes in HR in response to active stimulation.

### 3.4 Autonomic time course analysis

**Figure 3:**
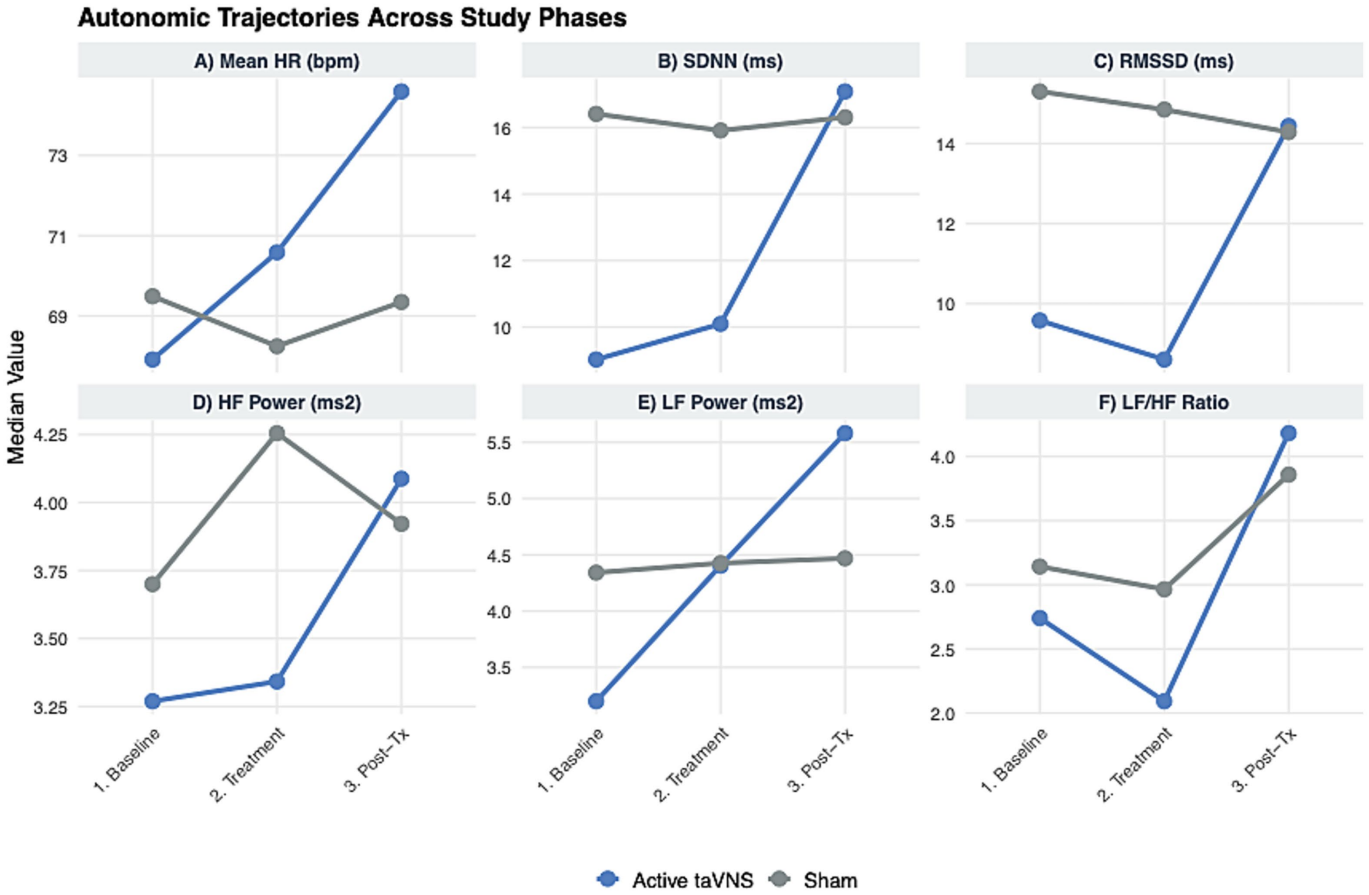
Longitudinal trajectories of heart rate variability metrics across study phases. A comparison of raw median values for six different cardiovascular autonomic indices between Active taVNS (green circles/lines) and Sham (gray circles/lines) conditions. Continuous HR data were acquired from eight individuals with Parkinson’s disease across three sequential epochs: 1. Baseline, 2. Treatment (15 min), and 3. Post-Tx washout. The six facets display distinct standard heart rate variability metrics: **(A)** Mean Heart Rate (bpm); **(B)** SDNN (ms); **(C)** RMSSD (ms); **(D)** HF Power (ms^2^); **(E)** LF Power (ms^2^); and **(F)** LF/HF Ratio. Individual data points are plotted as circles, and solid lines indicate the group trajectory. The blue lines represent active 30 Hz taVNS, and the gray lines represent sham (0 mA) stimulation. For detailed statistical analysis, please refer to Supplementary Materials.

To evaluate the temporal dynamics of autonomic modulation at rest, changes (Δ values) in continuous HRV metrics were analyzed using the Wilcoxon Signed-Rank test across three distinct intervals. (1) The Baseline to Treatment interval was assessed to quantify the immediate autonomic response during active versus sham stimulation. (2) The Baseline to Post-Treatment interval evaluated the cumulative autonomic shift encompassing both the stimulation and immediate washout periods. Finally, (3) the Treatment to Post-Treatment interval was analyzed to capture any physiological rebound effect following the cessation of the stimulus. No between-condition comparisons of these delta values met the conventional threshold for statistical significance (*p* < 0.05). However, during the transition from treatment to the post-treatment washout, active taVNS was associated with directionally consistent increases in several vagally mediated or global HRV metrics relative to sham, including RMSSD (*p* = 0.106), High-Frequency (HF) Power (*p* = 0.178), and SDNN (*p* = 0.178), along with a change in Heart Rate (*p* = 0.106; Figure 3).

### 3.5 Autonomic reactivity during Ewing battery

To evaluate whether autonomic responses after taVNS extended into periods of physiological stress, participants completed the Ewing Battery immediately following the post-treatment washout period. Autonomic reactivity was assessed using established clinical indices including the Valsalva ratio, the deep breathing heart rate response, and the orthostatic 30:15 ratio, with differences between conditions analyzed via the Wilcoxon Signed-Rank test. Comparisons of these primary reflex ratios did not reveal statistically significant differences between the active and sham conditions. During the deep breathing challenge, however, continuous HRV monitoring showed directionally consistent differences favoring greater vagally mediated HRV following active taVNS, including RMSSD (*p* = 0.142), pNN50 (*p* = 0.016), and HF power (*p* = 0.142), alongside a lower LF/HF ratio (*p* = 0.059). Although only pNN50 met the conventional nominal *p* < 0.05 threshold, the convergence of multiple HRV metrics in the same direction suggests a biologically plausible physiological pattern that warrants further investigation.

## 4 Discussion

The primary objective of this proof-of-concept pilot study was to evaluate whether continuous HRV monitoring and a standardized battery of autonomic reflex tests could be used to characterize acute CV autonomic responses to taVNS in patients with PD. Our findings support both the safety and feasibility of this methodological framework and suggest that it may be sensitive to real-time physiological changes. Across analyses, three exploratory patterns remerged: (1) baseline autonomic burden was associated with the magnitude of acute physiological response; (2) active taVNS was associated with directionally consistent changes in vagally mediated HRV metrics during the post-stimulation period; and (3) similar directional changes in vagally mediated HRV metrics were observed during the deep breathing challenge.

Our analysis of baseline clinical phenotypes provides preliminary insight into potential responder characteristics. Specifically, greater baseline autonomic symptom burden (higher COMPASS-31 scores) was associated with larger physiological responses to taVNS, including changes in HR and select HRV metrics. This finding suggests that responsiveness to taVNS may be influenced by baseline autonomic state, with individuals who present with greater dysautonomia exhibiting greater modulation of CV responses. One possible interpretation is that individuals with more impaired autonomic function may have increased capacity for modulation within an already dysregulated system. Alternatively, this pattern may reflect differences in central autonomic responsiveness or altered sensitivity of downstream efferent pathways. Mechanistically, these findings are consistent with the proposed effects of taVNS on central autonomic regulation (Carandina et al., 2021; Gharabaghi and Keute, 2025; Soltani et al., 2023). By increasing afferent input to the nucleus tractus solitarius, taVNS may modulate downstream autonomic output and influence coordination of heart rate and blood pressure regulation. However, given the small sample size, these findings should be interpreted as exploratory and hypothesis-generating, and further work is needed to determine whether baseline autonomic function can reliably predict treatment responsiveness.

Analysis of autonomic responses to taVNS at rest revealed directionally consistent changes in HRV metrics during the transition from stimulation to the post-treatment period. Specifically, active taVNS was associated with increases in RMSSD, HF power, and SDNN, suggesting a possible post-stimulation shift in autonomic state. These effects were most apparent during the post-treatment interval and were not observed in the sham condition, indicating a delayed or recovery-associated autonomic response. This pattern suggests that taVNS may modulate autonomic activity at rest, with effects that persist beyond the active stimulation period.

Complementary findings from the Ewing Battery indicate that this altered autonomic state may extend into periods of physiological challenge. While traditional indices of reflex function, such as the Valsalva ratio and 30:15 ratio, did not differ between conditions, directional increases in RMSSD and RSA magnitude during deep breathing were observed following active taVNS. These findings suggest that taVNS-related changes in autonomic regulation may extend past the 1-min post-stimulation period and influence responses to subsequent physiological challenges such as deep breathing. However, the functional implications of this shift are not immediately clear, particularly in relation to exercise. At face value, a shift toward parasympathetic dominance may appear counterintuitive, given that individuals with PD often exhibit a blunted heart rate response to exercise (Kanegusuku et al., 2022; Roberson et al., 2019; Sabino-Carvalho et al., 2018; Kanegusuku et al., 2016) and that exercise-induced increases in heart rate are primarily mediated by sympathetic activation (Gallo Júnior et al., 1989). However, autonomic function is not defined by the dominance of a single branch, but by the dynamic coordination between sympathetic and parasympathetic activity (Navarro, 2002). In PD, this coordination is disrupted, contributing to reduced autonomic flexibility and impaired cardiovascular responses (Rissardo et al., 2025; Ziemssen and Reichmann, 2010; Roberson et al., 2019; Ziemssen and Schmidt, 2005). Thus, a transient increase in parasympathetic activity may reflect improved regulatory capacity rather than simple vagal predominance. Whether this type of modulation supports more effective cardiovascular responses during exercise, including improved baroreflex function, requires further investigation.

Several limitations inherent to a proof-of-concept pilot study must be carefully acknowledged. Foremost, the small sample size (*N* = 8) limits statistical power and the generalizability of the findings. In addition, this study included a large number of endpoints and comparisons without correction for multiple testing, which increases the likelihood of type I error. Accordingly, isolated *p*-values near 0.05 should not be interpreted as definitive evidence of physiological efficacy, and all findings should be considered exploratory and hypothesis-generating. Additionally, the baseline duration for HRV recording was limited to 1 minute, which is shorter than the five-minute standard recommended for conventional HRV analysis (Circulation, 1996). While shorter recordings may be appropriate for capturing rapid, within-subject changes across controlled conditions, this limitation may affect the reliability of absolute HRV values and limit comparability with established normative data. The single-blind design represents an additional limitation, and future studies should employ a double-blinded study design. Finally, active stimulation was delivered 0.1 mA below each participant’s perceptual threshold to preserve blinding. Although this approach reduced the likelihood that participants could distinguish active from sham stimulation, sub-perceptual stimulation may have resulted in insufficient or inconsistent engagement of the intended neural target. This potential under-engagement could have contributed to the modest and statistically weak physiological effects observed in the present study and should be considered when designing future taVNS trials.

In conclusion, this study demonstrates that continuous HRV monitoring combined with standardized autonomic reflex testing is feasible for characterizing acute cardiovascular autonomic responses to taVNS in individuals with Parkinson’s disease. The observed physiological patterns suggest that active taVNS may be associated with directional changes in vagally mediated HRV metrics during the post-stimulation period and during autonomic challenge. These findings provide preliminary insight into potential responder characteristics and support the biological plausibility of acute autonomic modulation in PD. However, given the small sample size, multiple uncorrected comparisons, and exploratory design, these results should be viewed as hypothesis-generating. Future adequately powered studies are warranted to determine whether taVNS can reliably modulate CV autonomic regulation and whether repeated taVNS, applied in relation to therapeutic exercise, can meaningfully influence rehabilitation outcomes in this population.

## Data Availability

The raw data supporting the conclusions of this article will be made available by the authors, without undue reservation.

## References

[ref1] Ackland G. L. Patel A. B. U. Miller S. Gutierrez del Arroyo A. Thirugnanasambanthar J. Ravindran J. I. . (2025). Non-invasive vagus nerve stimulation and exercise capacity in healthy volunteers: a randomized trial. Eur. Heart J. 46, 1634–1644. doi: 10.1093/eurheartj/ehaf037, 39969124 PMC7617618

[ref2] Antonino D. Teixeira A. L. Maia-Lopes P. M. Souza M. C. Sabino-Carvalho J. L. Murray A. R. . (2017). Non-invasive vagus nerve stimulation acutely improves spontaneous cardiac baroreflex sensitivity in healthy young men: a randomized placebo-controlled trial. Brain Stimul. 10, 875–881. doi: 10.1016/j.brs.2017.05.006, 28566194

[ref3] Benarroch E. E. (2008). The arterial baroreflex: functional organization and involvement in neurologic disease. Neurology 71, 1733–1738. doi: 10.1212/01.wnl.0000335246.93495.92 19015490

[ref4] Carandina A. Rodrigues G. D. Di Francesco P. Filtz A. Bellocchi C. Furlan L. . (2021). Effects of transcutaneous auricular vagus nerve stimulation on cardiovascular autonomic control in health and disease. Auton. Neurosci. 236:102893. doi: 10.1016/j.autneu.2021.102893, 34649119

[ref5] Chen Z. Li G. Liu J. (2020). Autonomic dysfunction in Parkinson's disease: implications for pathophysiology, diagnosis, and treatment. Neurobiol. Dis. 134:104700. doi: 10.1016/j.nbd.2019.104700 31809788

[ref6] Heart rate variability: standards of measurement, physiological interpretation and clinical use. (1996). Task force of the European Society of Cardiology and the North American Society of Pacing and Electrophysiology. Circulation 93, 1043–1065. doi: 10.1161/01.CIR.93.5.1043, 8598068

[ref7] Clancy J. A. Mary D. A. Witte K. K. Greenwood J. P. Deuchars S. A. Deuchars J. (2014). Non-invasive Vagus nerve stimulation in healthy humans reduces sympathetic nerve activity. Brain Stimul. 7, 871–877. doi: 10.1016/j.brs.2014.07.031 25164906

[ref8] Collins L. Boddington L. Steffan P. J. McCormick D. (2021). Vagus nerve stimulation induces widespread cortical and behavioral activation. Curr. Biol. 31, 2088–98.e3. doi: 10.1016/j.cub.2021.02.049, 33740425

[ref9] Cuenca-Bermejo L. Almela P. Navarro-Zaragoza J. Fernández Villalba E. González-Cuello A. M. Laorden M. L. . (2021). Cardiac changes in Parkinson's disease: lessons from clinical and experimental evidence. Int. J. Mol. Sci. 22:13488. doi: 10.3390/ijms222413488, 34948285 PMC8705692

[ref10] Ernst M. Folkerts A. K. Gollan R. Lieker E. Caro-Valenzuela J. Adams A. . (2023). Physical exercise for people with Parkinson’s disease: a systematic review and network meta-analysis. Cochrane Database Syst. Rev. 2023:CD013856. doi: 10.1002/14651858.CD013856.pub2, 36602886 PMC9815433

[ref11] Ewing D. J. Clarke B. F. (1982). Diagnosis and management of diabetic autonomic neuropathy. Br. Med. J. (Clin. Res. Ed.) 285, 916–918. doi: 10.1136/bmj.285.6346.916, 6811067 PMC1500018

[ref12] Ewing D. J. Martyn C. N. Young R. J. Clarke B. F. (1985). The value of cardiovascular autonomic function tests: 10 years experience in diabetes. Diabetes Care 8, 491–498. doi: 10.2337/diacare.8.5.491, 4053936

[ref13] Feng Y. S. Yang S. D. Tan Z. X. Wang M. M. Xing Y. Dong F. . (2020). The benefits and mechanisms of exercise training for Parkinson's disease. Life Sci. 245:117345. doi: 10.1016/j.lfs.2020.117345, 31981631

[ref14] Forte G. Favieri F. Leemhuis E. De Martino M. L. Giannini A. M. De Gennaro L. . (2022). Ear your heart: transcutaneous auricular vagus nerve stimulation on heart rate variability in healthy young participants. PeerJ. 10:e14447. doi: 10.7717/peerj.14447, 36438582 PMC9686410

[ref15] Gallo Júnior L. Maciel B. C. Marin-Neto J. A. Martins L. E. (1989). Sympathetic and parasympathetic changes in heart rate control during dynamic exercise induced by endurance training in man. Braz. J. Med. Biol. Res. 22, 631–643. 2620172

[ref16] Gentile F. Giannoni A. Navari A. Degl'Innocenti E. Emdin M. Passino C. (2025). Acute right-sided transcutaneous vagus nerve stimulation improves cardio-vagal baroreflex gain in patients with chronic heart failure. Clin. Auton. Res. 35, 75–85. doi: 10.1007/s10286-024-01074-9, 39402309 PMC11937132

[ref17] Gharabaghi A. Keute M. (2025). Effects of stimulation site and protocol on autonomic responses to auricular vagus nerve stimulation. Front. Neurosci. 19:1692530. doi: 10.3389/fnins.2025.1692530, 41395270 PMC12698621

[ref18] Gilat M. Ginis P. Zoetewei D. De Vleeschhauwer J. Hulzinga F. D'Cruz N. . (2021). A systematic review on exercise and training-based interventions for freezing of gait in Parkinson's disease. NPJ Parkinsons Dis. 7:81. doi: 10.1038/s41531-021-00224-4, 34508083 PMC8433229

[ref19] Goldstein D. S. Holmes C. Li S. T. Bruce S. Metman L. V. Cannon R. O. 3rd. (2000). Cardiac sympathetic denervation in Parkinson disease. Ann. Intern. Med. 133, 338–347. doi: 10.7326/0003-4819-133-5-200009050-00009, 10979878

[ref20] Griffith G. Lamotte G. Mehta N. Fan P. Nikolich J. Springman V. . (2024). Chronotropic incompetence during exercise testing as a marker of autonomic dysfunction in individuals with early Parkinson's disease. J. Parkinsons Dis. 14, 121–133. doi: 10.3233/JPD-230006, 38189712 PMC10836543

[ref21] Hatik S. H. Asrlan M. Demirbilek Ö. Özden A. V. (2023). The effect of transcutaneous auricular vagus nerve stimulation on cycling ergometry and recovery in healthy young individuals. Brain Behav. 13:e3332. doi: 10.1002/brb3.3332, 37974551 PMC10726880

[ref22] Hirsch M. A. van Wegen E. E. H. Newman M. A. Heyn P. C. (2018). Exercise-induced increase in brain-derived neurotrophic factor in human Parkinson's disease: a systematic review and meta-analysis. Transl. Neurodegener. 7:7. doi: 10.1186/s40035-018-0112-1, 29568518 PMC5859548

[ref23] Johansson H. Hagströmer M. Grooten W. J. A. Franzén E. (2020). Exercise-induced neuroplasticity in Parkinson's disease: a metasynthesis of the literature. Neural Plast. 2020, 1–15. doi: 10.1155/2020/8961493, 32256559 PMC7079218

[ref24] Kamo H. Remz M. Mehta T. R. Burke R. M. Brooks A. Smiley A. . (2026). Five-year risk of cardiovascular events and falling in Parkinson's disease with orthostatic hypotension: a nationwide cohort study. Parkinsonism Relat. Disord. 144:108217. doi: 10.1016/j.parkreldis.2026.108217, 41637898

[ref25] Kanegusuku H. Cucato G. G. Longano P. Okamoto E. Piemonte M. E. P. Correia M. A. . (2022). Acute cardiovascular responses to self-selected intensity exercise in Parkinson's disease. Int. J. Sports Med. 43, 177–182. doi: 10.1055/a-1529-6480, 34380151

[ref26] Kanegusuku H. Silva-Batista C. Peçanha T. Nieuwboer A. Silva N. D. Jr. Costa L. A. . (2016). Blunted maximal and submaximal responses to cardiopulmonary exercise tests in patients with Parkinson disease. Arch. Phys. Med. Rehabil. 97, 720–725. doi: 10.1016/j.apmr.2015.12.020 26780469

[ref27] Kathia M. M. Duplea S. G. Bommarito J. C. Hinks A. Leake E. Shannon J. . (1985). High-intensity interval versus moderate-intensity continuous cycling training in Parkinson's disease: a randomized trial. J. Appl. Physiol. 137, 603–615. doi: 10.1152/japplphysiol.00219.2024, 39008618

[ref28] Keute M. Gharabaghi A. (2021). Brain plasticity and vagus nerve stimulation. Auton. Neurosci. 236:102876. doi: 10.1016/j.autneu.2021.102876, 34537681

[ref29] Kim J. S. Lee S. H. Oh Y. S. Park J. W. An J. Y. Park S. K. . (2016). Cardiovascular autonomic dysfunction in mild and advanced Parkinson's disease. J Mov Disord. 9, 97–103. doi: 10.14802/jmd.16001, 27020456 PMC4886202

[ref30] Li Y. Song H. L. Shen L. H. Wang Y. N. (2021). The efficacy and safety of moderate aerobic exercise for patients with Parkinson's disease: a systematic review and meta-analysis of randomized controlled trials. Ann. Palliat. Med. 10, 2638–2649. doi: 10.21037/apm-20-1661, 33549003

[ref31] Machado B. H. Mauad H. Chianca Júnior D. A. Haibara A. S. Colombari E. (1997). Autonomic processing of the cardiovascular reflexes in the nucleus tractus solitarii. Braz. J. Med. Biol. Res. 30, 533–535. doi: 10.1590/S0100-879X1997000400015 9251775

[ref32] Mak M. K. Wong-Yu I. S. Shen X. Chung C. L. (2017). Long-term effects of exercise and physical therapy in people with Parkinson disease. Nat. Rev. Neurol. 13, 689–703. doi: 10.1038/nrneurol.2017.128, 29027544

[ref33] Martin K. A. Papadoyannis E. S. Schiavo J. K. Fadaei S. S. Issa H. A. Song S. C. . (2024). Vagus nerve stimulation recruits the central cholinergic system to enhance perceptual learning. Nat. Neurosci. 27, 2152–2166. doi: 10.1038/s41593-024-01767-4, 39284963 PMC11932732

[ref34] Maule S. Milazzo V. Maule M. M. Di Stefano C. Milan A. Veglio F. (2012). Mortality and prognosis in patients with neurogenic orthostatic hypotension. Funct. Neurol. 27, 101–106. 23158582 PMC3812778

[ref35] Miyasato R. S. Silva-Batista C. Peçanha T. Low D. A. de Mello M. T. Piemonte M. E. P. . (2018). Cardiovascular responses during resistance exercise in patients with Parkinson disease. PM R 10, 1145–1152. doi: 10.1016/j.pmrj.2018.04.009, 29753113

[ref36] Navarro X. (2002). Physiology of the autonomic nervous system. Rev. Neurol. 35, 553–562. 12389173

[ref37] Palma J. A. Kaufmann H. (2020). Orthostatic hypotension in Parkinson disease. Clin. Geriatr. Med. 36, 53–67. doi: 10.1016/j.cger.2019.09.002, 31733702 PMC7029426

[ref38] Pfeiffer R. F. (2020). Autonomic dysfunction in Parkinson's disease. Neurotherapeutics 17, 1464–1479. doi: 10.1007/s13311-020-00897-4, 32789741 PMC7851208

[ref39] Ramos L. Watson J. Macalintal R. Ellis C. (2024). High-intensity exercise in community-based boxing improves functional limitations in individuals with Parkinson's disease. Int. J. Exerc. Sci. 17, 1493–1503. doi: 10.70252/IHKW5009, 39574811 PMC11581386

[ref40] Rissardo J. P. Gadelmawla A. F. Khalil I. Abdulgadir A. Bhatti K. S. Fornari Caprara A. L. (2025). Epidemiology of autonomic dysfunction in Parkinson's disease (review). Med Int (Lond). 5, 1–25. doi: 10.3892/mi.2025.267 41018272 PMC12464526

[ref41] Roberson K. B. Signorile J. F. Singer C. Jacobs K. A. Eltoukhy M. Ruta N. . (2019). Hemodynamic responses to an exercise stress test in Parkinson's disease patients without orthostatic hypotension. Appl. Physiol. Nutr. Metab. 44, 751–758. doi: 10.1139/apnm-2018-0638 30521353

[ref42] Sabino-Carvalho J. L. Teixeira A. L. Samora M. Daher M. Vianna L. C. (2018). Blunted cardiovascular responses to exercise in Parkinson's disease patients: role of the muscle metaboreflex. J. Neurophysiol. 120, 1516–1524. doi: 10.1152/jn.00308.2018, 29947592

[ref43] Sabino-Carvalho J. L. Vianna L. C. (2020). Altered cardiorespiratory regulation during exercise in patients with Parkinson's disease: a challenging non-motor feature. SAGE Open Med. 8:2050312120921603. doi: 10.1177/2050312120921603, 32435491 PMC7222646

[ref44] Schenkman M. Moore C. G. Kohrt W. M. Hall D. A. Delitto A. Comella C. L. . (2018). Effect of high-intensity treadmill exercise on motor symptoms in patients with de novo Parkinson disease: a phase 2 randomized clinical trial. JAMA Neurol. 75, 219–226. doi: 10.1001/jamaneurol.2017.3517, 29228079 PMC5838616

[ref45] Soltani D. Azizi B. Sima S. Tavakoli K. Hosseini Mohammadi N. S. Vahabie A.-H. . (2023). A systematic review of the effects of transcutaneous auricular vagus nerve stimulation on baroreflex sensitivity and heart rate variability in healthy subjects. Clin. Auton. Res. 33, 165–189. doi: 10.1007/s10286-023-00938-w 37119426

[ref46] Urbin M. A. Lafe C. W. Simpson T. W. Wittenberg G. F. Chandrasekaran B. Weber D. J. (2021). Electrical stimulation of the external ear acutely activates noradrenergic mechanisms in humans. Brain Stimul. 14, 990–1001. doi: 10.1016/j.brs.2021.06.002, 34154980

[ref47] White D. W. Raven P. B. (2014). Autonomic neural control of heart rate during dynamic exercise: revisited. J. Physiol. 592, 2491–2500. doi: 10.1113/jphysiol.2014.271858, 24756637 PMC4080933

[ref48] Yap J. Y. Y. Keatch C. Lambert E. Woods W. Stoddart P. R. Kameneva T. (2020). Critical review of transcutaneous Vagus nerve stimulation: challenges for translation to clinical practice. Front. Neurosci. 14:284. doi: 10.3389/fnins.2020.00284 32410932 PMC7199464

[ref49] Yokota H. Edama M. Hirabayashi R. Sekine C. Otsuru N. Saito K. . (2022). Effects of stimulus frequency, intensity, and sex on the autonomic response to transcutaneous vagus nerve stimulation. Brain Sci. 12:1038. 36009101 10.3390/brainsci12081038 PMC9405815

[ref50] Yoshida Y. Okayama S. Fujihara D. Taniyama M. Yamada A. Fukui M. . (2025). Effects of transcutaneous auricular vagus nerve stimulation on hemodynamics and autonomic function during exercise stress tests in healthy volunteers. Circ Rep. 7, 315–322. doi: 10.1253/circrep.CR-24-0136, 40352124 PMC12061502

[ref51] Ziemssen T. Reichmann H. (2010). Cardiovascular autonomic dysfunction in Parkinson's disease. J. Neurol. Sci. 289, 74–80. doi: 10.1016/j.jns.2009.08.031, 19740484

[ref52] Ziemssen T. Schmidt C. (2005). Dysautonomia in Parkinson's disease. Psychopharmakotherapie 12, 6–12.

