# Supplementary material for: Modulating autonomic nervous system activity with transcutaneous auricular vagus nerve stimulation in Parkinson’s disease: a proof of concept study
Source: Front Neurosci. 2026 Jul 2;20:1830260. doi: 10.3389/fnins.2026.1830260 (PMC13372895; doi:10.3389/fnins.2026.1830260)
Supplement: Supplementary file 1 [file Table_1.DOCX]

Supplementary Material

# Supplementary Data

Supplementary Table 1: Physiological Responses to Active vs. Sham taVNS

| **Variable** | **Active taVNS (Median [IQR])** | **Sham (Median [IQR])** | **P-Value** |
| --- | --- | --- | --- |
| Valsalva Ratio | 1.23 [1.17 - 1.29] | 1.26 [1.23 - 1.34] | 0.529 |
| Valsalva HR Max (bpm) | 77.64 [72.63 - 81.09] | 77.54 [74.48 - 83.94] | 0.208 |
| Valsalva HR Min (bpm) | 62.08 [59.55 - 63.27] | 62.01 [57.59 - 65.37] | 1.000 |
| Deep Breathing HR Response | 8.89 [6.29 - 9.85] | 6.88 [5.93 - 8.85] | 0.675 |
| Deep Breathing Mean HR | 60.97 [58.82 - 69.03] | 64.00 [62.55 - 67.09] | 1.000 |
| Deep Breathing RMSSD (ms) | 41.09 [20.38 - 47.88] | 27.83 [19.97 - 32.78] | 0.142 † |
| Deep Breathing SDNN (ms) | 55.83 [28.09 - 66.66] | 41.95 [28.71 - 46.02] | 0.295 |
| Deep Breathing E/I Ratio | 1.16 [1.10 - 1.19] | 1.12 [1.09 - 1.15] | 0.402 |
| Deep Breathing PNS Index | 0.36 [-0.97 - 0.53] | -0.40 [-0.90 - 0.01] | 0.208 |
| Deep Breathing RSA Magnitude | 48.59 [22.91 - 49.94] | 37.28 [26.91 - 39.57] | 0.142 † |
| Orthostatic 30:15 Ratio | 1.11 [1.10 - 1.12] | 1.15 [1.10 - 1.17] | 1.000 |
| Orthostatic Supine HR | 61.78 [58.45 - 67.50] | 61.62 [60.49 - 65.78] | 0.787 |
| Orthostatic Stand HR | 76.74 [69.23 - 82.20] | 73.96 [70.08 - 74.52] | 0.281 |
| Orthostatic Supine PNS | -0.62 [-0.77 - -0.04] | -0.50 [-0.60 - -0.41] | 1.000 |
| Orthostatic Stand PNS | -1.76 [-1.79 - -1.08] | -1.39 [-1.41 - -1.32] | 0.281 |
| Orthostatic Supine RMSSD | 24.67 [12.64 - 26.77] | 18.52 [15.41 - 20.32] | 0.787 |
| Orthostatic Stand RMSSD | 12.16 [6.44 - 13.23] | 11.40 [9.63 - 15.74] | 0.418 |
| Orthostatic Supine LF | 105.13 [73.27 - 297.89] | 142.04 [108.80 - 225.18] | 0.590 |
| Orthostatic Stand LF | 92.13 [14.95 - 174.41] | 66.51 [23.98 - 232.09] | 0.281 |
| Orthostatic Supine HF | 272.28 [50.32 - 326.96] | 123.21 [87.58 - 142.37] | 0.281 |
| Orthostatic Stand HF | 55.98 [15.04 - 78.04] | 45.09 [32.22 - 75.30] | 1.000 |
| † Approaching statistical significance (p < 0.20). | | | |

Supplementary Table 2: Spearman Correlations between Clinical Predictors and taVNS Reactivity (Deltas)

| **Variable** | **COMPASS-31 Total** | **SCOPA-AUT Total** | **GLTQ** | **IPAQ METS** | **MDS-UPDRS Motor** |
| --- | --- | --- | --- | --- | --- |
| Valsalva Ratio | -0.26 (p=0.623) | 0.29 (p=0.577) | -0.20 (p=0.704) | 0.03 (p=0.957) | 0.20 (p=0.700) |
| Valsalva HR Max (bpm) | -0.66 (p=0.156) | 0.06 (p=0.913) | -0.20 (p=0.704) | -0.31 (p=0.544) | 0.55 (p=0.257) |
| Valsalva HR Min (bpm) | -0.14 (p=0.787) | -0.12 (p=0.827) | 0.20 (p=0.704) | -0.20 (p=0.704) | 0.52 (p=0.288) |
| Deep Breathing HR Response | 0.03 (p=0.957) | -0.12 (p=0.827) | 0.09 (p=0.872) | -0.26 (p=0.623) | 0.38 (p=0.461) |
| Deep Breathing Mean HR | -0.37 (p=0.468) | -0.17 (p=0.742) | -0.09 (p=0.872) | -0.26 (p=0.623) | 0.70 (p=0.125) |
| Deep Breathing RMSSD (ms) | 0.66 (p=0.156) | -0.09 (p=0.870) | -0.09 (p=0.872) | -0.09 (p=0.872) | -0.17 (p=0.742) |
| Deep Breathing SDNN (ms) | 0.60 (p=0.208) | 0.38 (p=0.461) | 0.26 (p=0.623) | 0.26 (p=0.623) | -0.23 (p=0.658) |
| Deep Breathing E/I Ratio | -0.09 (p=0.872) | -0.38 (p=0.461) | -0.09 (p=0.872) | -0.43 (p=0.397) | 0.23 (p=0.658) |
| Deep Breathing PNS Index | 0.54 (p=0.266) | 0.23 (p=0.658) | 0.20 (p=0.704) | 0.31 (p=0.544) | -0.61 (p=0.200) |
| Deep Breathing RSA Magnitude | 0.14 (p=0.787) | -0.20 (p=0.700) | 0.66 (p=0.156) | 0.54 (p=0.266) | *-0.75 (p=0.084)†* |
| Orthostatic 30:15 Ratio | 0.40 (p=0.505) | 0.41 (p=0.493) | 0.70 (p=0.188) | **0.90 (p=0.037)*** | -0.36 (p=0.553) |
| Orthostatic Supine HR | -0.20 (p=0.747) | 0.10 (p=0.870) | 0.10 (p=0.873) | 0.30 (p=0.624) | 0.21 (p=0.741) |
| Orthostatic Stand HR | -0.10 (p=0.873) | -0.05 (p=0.935) | 0.20 (p=0.747) | 0.50 (p=0.391) | -0.21 (p=0.741) |
| Orthostatic Supine PNS | 0.50 (p=0.391) | 0.10 (p=0.870) | 0.20 (p=0.747) | 0.10 (p=0.873) | -0.46 (p=0.434) |
| Orthostatic Stand PNS | 0.30 (p=0.624) | 0.41 (p=0.493) | 0.00 (p=1.000) | -0.30 (p=0.624) | 0.36 (p=0.553) |
| Orthostatic Supine RMSSD | 0.20 (p=0.747) | -0.15 (p=0.805) | 0.50 (p=0.391) | 0.30 (p=0.624) | -0.62 (p=0.269) |
| Orthostatic Stand RMSSD | 0.10 (p=0.873) | 0.05 (p=0.935) | -0.20 (p=0.747) | -0.50 (p=0.391) | 0.21 (p=0.741) |
| Orthostatic Supine LF | -0.80 (p=0.104) | -0.56 (p=0.322) | 0.10 (p=0.873) | -0.30 (p=0.624) | 0.21 (p=0.741) |
| Orthostatic Stand LF | 0.30 (p=0.624) | 0.56 (p=0.322) | -0.60 (p=0.285) | -0.70 (p=0.188) | *0.82 (p=0.089)†* |
| Orthostatic Supine HF | 0.20 (p=0.747) | -0.15 (p=0.805) | 0.50 (p=0.391) | 0.30 (p=0.624) | -0.62 (p=0.269) |
| Orthostatic Stand HF | -0.20 (p=0.747) | -0.21 (p=0.741) | 0.10 (p=0.873) | -0.30 (p=0.624) | 0.05 (p=0.935) |
| * Statistically significant (p < 0.05). Highlighted in Green. | | | | | |
| † Approaching statistical significance (p < 0.10). Highlighted in Yellow. | | | | | |
| Data presented as: Spearman's rho (p-value). | | | | | |

Supplementary Table 3: Acute Resting State Physiological Responses Across Treatment Phases

| **Phase** | **Variable** | **Active taVNS (Median [IQR])** | **Sham (Median [IQR])** | **P-Value** |
| --- | --- | --- | --- | --- |
| 1. Baseline to Treatment | PNS Index | -0.03 [-0.09 - 0.08] | -0.01 [-0.11 - 0.09] | 0.590 |
|  | SNS Index | -0.81 [-0.99 - -0.76] | -0.55 [-1.53 - -0.17] | 0.787 |
|  | Stress Index | -6.02 [-6.40 - -4.03] | -2.87 [-11.08 - -1.05] | 0.787 |
|  | Mean RR (ms) | 0.01 [-22.91 - 18.41] | 17.10 [7.01 - 19.76] | 1.000 |
|  | SDNN (ms) | -1.04 [-1.11 - -0.90] | -0.50 [-0.62 - 1.10] | 0.787 |
|  | Heart Rate (bpm) | -0.00 [-1.56 - 1.75] | -0.54 [-1.35 - 4.32] | 0.281 |
|  | RMSSD (ms) | -0.66 [-0.97 - 0.04] | -1.19 [-3.83 - -0.45] | 0.281 |
|  | pNN50 (%) | 0.00 [0.00 - 0.00] | 0.00 [0.00 - 0.62] | 0.423 |
|  | LF Power | 0.72 [-0.33 - 0.79] | 0.55 [0.19 - 0.65] | 0.281 |
|  | HF Power | 0.02 [-0.02 - 0.07] | -0.08 [-0.59 - 0.48] | 0.590 |
|  | LF/HF Ratio | 0.23 [-1.08 - 1.13] | 0.49 [-1.73 - 0.69] | *0.106 †* |
| 2. Baseline to Post-Treatment | PNS Index | -0.14 [-0.21 - -0.13] | -0.36 [-0.44 - -0.14] | 0.418 |
|  | SNS Index | 0.42 [-0.25 - 0.63] | 0.04 [-0.71 - 0.13] | 0.787 |
|  | Stress Index | 1.20 [-2.62 - 2.51] | -1.04 [-4.38 - 0.06] | 0.787 |
|  | Mean RR (ms) | -28.17 [-35.72 - -26.76] | -20.10 [-59.30 - -12.14] | 0.590 |
|  | SDNN (ms) | 3.03 [2.51 - 4.39] | 0.83 [-0.10 - 0.93] | 0.281 |
|  | Heart Rate (bpm) | 2.40 [1.83 - 3.45] | 0.99 [-0.21 - 1.50] | 0.418 |
|  | RMSSD (ms) | 1.17 [-0.49 - 2.70] | -1.67 [-6.00 - -1.02] | *0.178 †* |
|  | pNN50 (%) | 0.00 [0.00 - 1.59] | 0.00 [0.00 - 0.00] | 0.423 |
|  | LF Power | 0.57 [0.32 - 1.43] | 0.32 [0.03 - 0.84] | 0.281 |
|  | HF Power | 0.40 [-0.28 - 0.66] | -0.65 [-0.67 - 0.05] | 0.281 |
|  | LF/HF Ratio | 3.93 [-0.08 - 4.80] | 0.66 [-0.84 - 1.18] | 0.418 |
| 3. Treatment to Post-Treatment | PNS Index | -0.23 [-0.28 - -0.12] | -0.24 [-0.25 - -0.08] | 0.787 |
|  | SNS Index | 0.51 [0.32 - 1.23] | 0.68 [0.63 - 0.75] | 0.590 |
|  | Stress Index | 1.41 [-0.08 - 8.52] | 2.93 [1.24 - 3.67] | 0.787 |
|  | Mean RR (ms) | -45.17 [-45.75 - -35.73] | -29.25 [-42.94 - -4.28] | 0.590 |
|  | SDNN (ms) | 5.43 [0.29 - 9.54] | 1.45 [0.39 - 2.75] | *0.178 †* |
|  | Heart Rate (bpm) | 3.68 [3.45 - 3.97] | 0.33 [-0.14 - 2.35] | *0.106 †* |
|  | RMSSD (ms) | 1.88 [1.13 - 3.04] | -0.48 [-0.56 - -0.48] | *0.106 †* |
|  | pNN50 (%) | 0.00 [0.00 - 0.00] | 0.00 [0.00 - 0.47] | 0.423 |
|  | LF Power | 0.81 [0.22 - 0.90] | 0.20 [-0.16 - 0.58] | 0.590 |
|  | HF Power | 0.56 [-0.30 - 0.68] | -0.43 [-0.43 - -0.09] | *0.178 †* |
|  | LF/HF Ratio | 0.83 [-1.21 - 3.70] | 0.89 [0.69 - 4.14] | 0.787 |
| * Statistically significant (p < 0.05). Highlighted in Green. | | | | |
| † Approaching statistical significance (p < 0.20). Highlighted in Yellow. | | | | |
| Data analyzed via Wilcoxon Signed-Rank Test. | | | | |

Supplementary Table 4: Correlations Between Baseline Clinical Scores and Acute taVNS Physiological Response

| **Variable** | **COMPASS-31 Total** | **SCOPA-AUT Total** | **GLTQ** | **IPAQ METS** | **MDS-UPDRS Motor** |
| --- | --- | --- | --- | --- | --- |
| Delta PNS Index | **-0.90 (p=0.037)*** | -0.30 (p=0.624) | 0.10 (p=0.873) | -0.30 (p=0.624) | 0.10 (p=0.873) |
| Delta SNS Index | *0.70 (p=0.188)†* | 0.40 (p=0.505) | *0.80 (p=0.104)†* | **0.90 (p=0.037)*** | *-0.70 (p=0.188)†* |
| Delta Stress Index | *0.70 (p=0.188)†* | 0.40 (p=0.505) | *0.80 (p=0.104)†* | **0.90 (p=0.037)*** | *-0.70 (p=0.188)†* |
| Delta Mean RR (ms) | **-0.90 (p=0.037)*** | -0.30 (p=0.624) | 0.10 (p=0.873) | -0.30 (p=0.624) | 0.10 (p=0.873) |
| Delta SDNN (ms) | 0.00 (p=1.000) | 0.00 (p=1.000) | -0.20 (p=0.747) | -0.40 (p=0.505) | *0.80 (p=0.104)†* |
| Delta Heart Rate (bpm) | **0.90 (p=0.037)*** | 0.30 (p=0.624) | -0.10 (p=0.873) | 0.30 (p=0.624) | -0.10 (p=0.873) |
| Delta RMSSD (ms) | -0.30 (p=0.624) | -0.60 (p=0.285) | -0.20 (p=0.747) | -0.50 (p=0.391) | 0.30 (p=0.624) |
| Delta pNN50 (%) | -0.67 (p=0.215) | -0.22 (p=0.718) | 0.22 (p=0.718) | -0.22 (p=0.718) | 0.22 (p=0.718) |
| Delta LF Power | -0.30 (p=0.624) | -0.60 (p=0.285) | *-0.80 (p=0.104)†* | **-0.90 (p=0.037)*** | *0.70 (p=0.188)†* |
| Delta HF Power | *-0.70 (p=0.188)†* | 0.10 (p=0.873) | 0.30 (p=0.624) | -0.10 (p=0.873) | 0.30 (p=0.624) |
| Delta LF/HF Ratio | -0.30 (p=0.624) | -0.60 (p=0.285) | *-0.80 (p=0.104)†* | **-0.90 (p=0.037)*** | *0.70 (p=0.188)†* |
| * Statistically significant (p < 0.05). Highlighted in Green. | | | | | |
| † Approaching statistical significance (p < 0.20). Highlighted in Yellow. | | | | | |
| Data presented as: Spearman's rho (p-value). | | | | | |

Supplementary Table 5: Linear Mixed-Effects Model Results (Condition x Time Interaction)

| **Variable** | **Condition (P)** | **Time (P)** | **Interaction (P)** |
| --- | --- | --- | --- |
| PNS Index | 0.475 | 0.173 † | 0.980 |
| SNS Index | 0.917 | 0.024 * | 0.927 |
| Stress Index | 0.868 | 0.042 * | 0.896 |
| Mean RR (ms) | 0.538 | 0.079 † | 0.851 |
| SDNN (ms) | 0.756 | 0.072 † | 0.335 |
| RMSSD (ms) | 0.575 | 0.539 | 0.362 |
| pNN50 (%) | 0.233 | 0.269 | 0.548 |
| LF Power | 0.166 † | 0.177 † | 0.209 |
| HF Power | 0.140 † | 0.975 | 0.454 |
| LF/HF Ratio | 0.915 | 0.876 | 0.234 |
| * Statistically significant interaction (p < 0.05). | | | |
| † Approaching statistical significance (p < 0.20). | | | |
| Note: Interaction (Condition:Time) indicates if the trajectory of change differs between Active and Sham. | | | |

Supplementary Table 6: Active vs. Sham HRV Responses During the Ewing Battery

| **Ewing Test** | **Variable** | **Active taVNS (Median [IQR])** | **Sham (Median [IQR])** | **P-Value** |
| --- | --- | --- | --- | --- |
| 1. Valsalva Maneuver | PNS Index | -0.93 [-1.42 - -0.70] | -1.07 [-1.35 - -0.83] | 1.000 |
|  | SNS Index | 0.99 [0.38 - 1.95] | 1.01 [0.84 - 2.28] | 0.529 |
|  | Stress Index | 12.28 [10.05 - 15.46] | 14.16 [11.94 - 20.00] | 0.295 |
|  | Mean RR (ms) | 837.99 [809.38 - 894.90] | 860.53 [849.93 - 880.84] | 0.675 |
|  | SDNN (ms) | 41.31 [32.32 - 47.72] | 31.72 [21.61 - 38.98] | 0.675 |
|  | Mean HR (bpm) | 71.61 [67.11 - 74.14] | 69.73 [68.12 - 70.59] | 0.529 |
|  | RMSSD (ms) | 20.25 [16.46 - 33.73] | 17.12 [12.67 - 22.22] | 0.402 |
|  | pNN50 (%) | 2.97 [0.71 - 17.98] | 0.71 [0.00 - 2.56] | 0.584 |
|  | LF Power | 6.85 [6.39 - 6.95] | 6.04 [5.21 - 7.03] | 0.402 |
|  | HF Power | 4.57 [3.67 - 5.61] | 5.00 [3.92 - 5.18] | 0.529 |
|  | LF/HF Ratio | 10.70 [3.85 - 16.06] | 5.94 [4.76 - 7.82] | 0.675 |
| 2. Deep Breathing | PNS Index | 0.36 [-0.97 - 0.53] | -0.60 [-0.98 - -0.39] | 0.208 |
|  | SNS Index | -0.42 [-0.55 - 2.51] | 0.38 [0.09 - 1.48] | 0.834 |
|  | Stress Index | 8.86 [7.48 - 22.42] | 11.24 [9.02 - 17.04] | 0.675 |
|  | Mean RR (ms) | 985.39 [871.56 - 1020.02] | 905.13 [853.91 - 943.31] | 0.402 |
|  | SDNN (ms) | 55.83 [28.09 - 66.67] | 41.95 [28.71 - 46.02] | 0.295 |
|  | Mean HR (bpm) | 60.96 [58.82 - 69.02] | 66.32 [63.61 - 70.29] | 0.529 |
|  | RMSSD (ms) | 41.09 [20.39 - 47.88] | 27.83 [19.97 - 32.78] | *0.142 †* |
|  | pNN50 (%) | 20.69 [4.74 - 24.35] | 7.11 [2.28 - 10.53] | *0.106 †* |
|  | LF Power | 7.80 [5.99 - 7.90] | 7.27 [6.59 - 7.39] | 1.000 |
|  | HF Power | 5.33 [4.67 - 5.87] | 4.50 [3.94 - 4.60] | *0.142 †* |
|  | LF/HF Ratio | 6.72 [3.26 - 10.25] | 11.41 [6.24 - 16.90] | *0.059 †* |
| 3. Orthostatic (Supine) | PNS Index | -0.33 [-0.73 - 0.11] | -0.46 [-0.58 - -0.26] | 1.000 |
|  | SNS Index | 0.31 [0.00 - 1.27] | 0.53 [0.33 - 0.89] | 1.000 |
|  | Stress Index | 13.73 [12.34 - 18.81] | 14.53 [13.73 - 15.68] | 1.000 |
|  | Mean RR (ms) | 998.92 [909.51 - 1036.47] | 982.84 [928.34 - 1010.50] | 1.000 |
|  | SDNN (ms) | 21.71 [16.75 - 29.96] | 22.09 [18.38 - 26.13] | 1.000 |
|  | Mean HR (bpm) | 60.11 [57.89 - 66.07] | 61.05 [59.38 - 64.68] | 1.000 |
|  | RMSSD (ms) | 19.90 [13.27 - 26.24] | 17.91 [15.84 - 19.89] | 0.834 |
|  | pNN50 (%) | 2.11 [0.19 - 4.22] | 0.62 [0.52 - 2.20] | 0.402 |
|  | LF Power | 5.28 [5.09 - 6.19] | 5.71 [5.29 - 6.25] | 0.675 |
|  | HF Power | 4.54 [3.75 - 5.32] | 4.68 [4.51 - 4.83] | 0.834 |
|  | LF/HF Ratio | 4.08 [2.38 - 4.25] | 3.22 [2.70 - 4.50] | 0.675 |
| 4. Orthostatic (Stand) | PNS Index | -1.78 [-1.82 - -1.25] | -1.40 [-1.43 - -1.34] | 0.295 |
|  | SNS Index | 3.02 [2.13 - 4.30] | 2.59 [1.79 - 2.73] | 0.402 |
|  | Stress Index | 22.81 [17.52 - 31.47] | 22.99 [16.36 - 23.30] | 0.529 |
|  | Mean RR (ms) | 773.83 [738.89 - 845.52] | 808.23 [776.62 - 844.92] | 0.295 |
|  | SDNN (ms) | 14.23 [9.36 - 23.20] | 14.41 [10.39 - 24.24] | 0.529 |
|  | Mean HR (bpm) | 77.54 [71.10 - 81.24] | 74.24 [71.05 - 77.29] | 0.295 |
|  | RMSSD (ms) | 9.30 [5.55 - 12.96] | 10.51 [7.40 - 14.65] | 0.529 |
|  | pNN50 (%) | 0.00 [0.00 - 0.17] | 0.00 [0.00 - 0.23] | 1.000 |
|  | LF Power | 4.51 [2.67 - 5.72] | 4.72 [3.38 - 5.84] | *0.059 †* |
|  | HF Power | 3.12 [1.60 - 4.04] | 3.62 [2.74 - 3.89] | *0.142 †* |
|  | LF/HF Ratio | 6.11 [3.96 - 8.98] | 6.01 [4.27 - 7.28] | 0.675 |
| * Statistically significant (p < 0.05). Highlighted in Green. | | | | |
| † Approaching statistical significance (p < 0.20). Highlighted in Yellow. | | | | |
| Data analyzed via Wilcoxon Signed-Rank Test. | | | | |

Supplementary Table 7: Correlations Between Clinical Severity and taVNS-Induced HRV Changes During Autonomic Challenges

| **Ewing Test** | **Variable** | **COMPASS-31 Total** | **SCOPA-AUT Total** | **GLTQ** | **IPAQ METS** | **MDS-UPDRS Motor** |
| --- | --- | --- | --- | --- | --- | --- |
| 1. Valsalva Maneuver | Delta PNS Index | 0.14 (p=0.787) | 0.03 (p=0.957) | -0.20 (p=0.704) | -0.31 (p=0.544) | 0.06 (p=0.913) |
|  | Delta SNS Index | -0.03 (p=0.957) | -0.43 (p=0.389) | 0.09 (p=0.872) | 0.09 (p=0.872) | -0.17 (p=0.742) |
|  | Delta Stress Index | -0.09 (p=0.872) | -0.58 (p=0.228) | 0.20 (p=0.704) | 0.14 (p=0.787) | -0.41 (p=0.425) |
|  | Delta Mean RR (ms) | 0.14 (p=0.787) | 0.03 (p=0.957) | -0.20 (p=0.704) | -0.31 (p=0.544) | 0.06 (p=0.913) |
|  | Delta SDNN (ms) | -0.20 (p=0.704) | 0.29 (p=0.577) | 0.14 (p=0.787) | 0.03 (p=0.957) | 0.09 (p=0.870) |
|  | Delta Mean HR (bpm) | -0.14 (p=0.787) | -0.03 (p=0.957) | 0.20 (p=0.704) | 0.31 (p=0.544) | -0.06 (p=0.913) |
|  | Delta RMSSD (ms) | 0.03 (p=0.957) | 0.43 (p=0.389) | -0.09 (p=0.872) | -0.09 (p=0.872) | 0.17 (p=0.742) |
|  | Delta pNN50 (%) | 0.20 (p=0.700) | 0.16 (p=0.759) | -0.12 (p=0.827) | -0.23 (p=0.658) | 0.13 (p=0.803) |
|  | Delta LF Power | 0.03 (p=0.957) | 0.14 (p=0.784) | **-0.83 (p=0.042)*** | -0.71 (p=0.111) | 0.52 (p=0.288) |
|  | Delta HF Power | 0.43 (p=0.397) | 0.32 (p=0.538) | -0.20 (p=0.704) | 0.20 (p=0.704) | -0.61 (p=0.200) |
|  | Delta LF/HF Ratio | -0.49 (p=0.329) | -0.52 (p=0.288) | *-0.77 (p=0.072)†* | **-0.94 (p=0.005)*** | 0.70 (p=0.125) |
| 2. Deep Breathing | Delta PNS Index | 0.66 (p=0.156) | 0.20 (p=0.700) | 0.31 (p=0.544) | 0.54 (p=0.266) | **-0.81 (p=0.050)*** |
|  | Delta SNS Index | -0.54 (p=0.266) | -0.23 (p=0.658) | -0.20 (p=0.704) | -0.31 (p=0.544) | 0.61 (p=0.200) |
|  | Delta Stress Index | -0.26 (p=0.623) | 0.03 (p=0.957) | -0.31 (p=0.544) | -0.20 (p=0.704) | 0.46 (p=0.354) |
|  | Delta Mean RR (ms) | 0.49 (p=0.329) | 0.14 (p=0.784) | 0.20 (p=0.704) | 0.49 (p=0.329) | **-0.90 (p=0.015)*** |
|  | Delta SDNN (ms) | 0.60 (p=0.208) | 0.38 (p=0.461) | 0.26 (p=0.623) | 0.26 (p=0.623) | -0.23 (p=0.658) |
|  | Delta Mean HR (bpm) | -0.49 (p=0.329) | -0.14 (p=0.784) | -0.20 (p=0.704) | -0.49 (p=0.329) | **0.90 (p=0.015)*** |
|  | Delta RMSSD (ms) | 0.66 (p=0.156) | -0.06 (p=0.913) | 0.20 (p=0.704) | 0.31 (p=0.544) | -0.55 (p=0.257) |
|  | Delta pNN50 (%) | 0.71 (p=0.111) | 0.29 (p=0.577) | 0.14 (p=0.787) | 0.37 (p=0.468) | -0.67 (p=0.148) |
|  | Delta LF Power | 0.26 (p=0.623) | -0.03 (p=0.957) | 0.31 (p=0.544) | 0.20 (p=0.704) | -0.46 (p=0.354) |
|  | Delta HF Power | -0.09 (p=0.872) | -0.38 (p=0.461) | -0.09 (p=0.872) | -0.43 (p=0.397) | 0.23 (p=0.658) |
|  | Delta LF/HF Ratio | 0.20 (p=0.704) | 0.32 (p=0.538) | -0.09 (p=0.872) | 0.26 (p=0.623) | -0.06 (p=0.913) |
| 3. Orthostatic (Supine) | Delta PNS Index | 0.49 (p=0.329) | 0.12 (p=0.827) | 0.09 (p=0.872) | 0.09 (p=0.872) | -0.38 (p=0.461) |
|  | Delta SNS Index | -0.26 (p=0.623) | 0.03 (p=0.957) | -0.31 (p=0.544) | -0.20 (p=0.704) | 0.46 (p=0.354) |
|  | Delta Stress Index | -0.31 (p=0.544) | -0.09 (p=0.870) | -0.43 (p=0.397) | -0.43 (p=0.397) | 0.70 (p=0.125) |
|  | Delta Mean RR (ms) | 0.26 (p=0.623) | -0.06 (p=0.913) | -0.14 (p=0.787) | -0.20 (p=0.704) | -0.23 (p=0.658) |
|  | Delta SDNN (ms) | 0.09 (p=0.872) | -0.06 (p=0.913) | 0.49 (p=0.329) | 0.54 (p=0.266) | **-0.93 (p=0.008)*** |
|  | Delta Mean HR (bpm) | -0.26 (p=0.623) | 0.06 (p=0.913) | 0.14 (p=0.787) | 0.20 (p=0.704) | 0.23 (p=0.658) |
|  | Delta RMSSD (ms) | 0.31 (p=0.544) | 0.09 (p=0.870) | 0.43 (p=0.397) | 0.43 (p=0.397) | -0.70 (p=0.125) |
|  | Delta pNN50 (%) | 0.31 (p=0.544) | 0.09 (p=0.870) | 0.43 (p=0.397) | 0.43 (p=0.397) | -0.70 (p=0.125) |
|  | Delta LF Power | -0.31 (p=0.544) | -0.52 (p=0.288) | 0.49 (p=0.329) | 0.37 (p=0.468) | **-0.84 (p=0.036)*** |
|  | Delta HF Power | 0.37 (p=0.468) | 0.17 (p=0.742) | 0.09 (p=0.872) | 0.26 (p=0.623) | -0.70 (p=0.125) |
|  | Delta LF/HF Ratio | -0.31 (p=0.544) | -0.46 (p=0.354) | 0.54 (p=0.266) | 0.49 (p=0.329) | *-0.75 (p=0.084)†* |
| 4. Orthostatic (Stand) | Delta PNS Index | 0.26 (p=0.623) | 0.29 (p=0.577) | -0.03 (p=0.957) | -0.14 (p=0.787) | 0.20 (p=0.700) |
|  | Delta SNS Index | -0.60 (p=0.208) | -0.06 (p=0.913) | 0.09 (p=0.872) | 0.09 (p=0.872) | 0.20 (p=0.700) |
|  | Delta Stress Index | -0.49 (p=0.329) | 0.14 (p=0.784) | 0.03 (p=0.957) | 0.14 (p=0.787) | 0.12 (p=0.827) |
|  | Delta Mean RR (ms) | 0.49 (p=0.329) | 0.12 (p=0.827) | 0.09 (p=0.872) | 0.09 (p=0.872) | -0.38 (p=0.461) |
|  | Delta SDNN (ms) | 0.20 (p=0.704) | -0.43 (p=0.389) | -0.54 (p=0.266) | -0.66 (p=0.156) | 0.12 (p=0.827) |
|  | Delta Mean HR (bpm) | -0.14 (p=0.787) | -0.03 (p=0.957) | 0.20 (p=0.704) | 0.31 (p=0.544) | -0.06 (p=0.913) |
|  | Delta RMSSD (ms) | 0.14 (p=0.787) | 0.03 (p=0.957) | -0.20 (p=0.704) | -0.31 (p=0.544) | 0.06 (p=0.913) |
|  | Delta pNN50 (%) | -0.51 (p=0.305) | -0.17 (p=0.745) | 0.17 (p=0.749) | -0.17 (p=0.749) | 0.17 (p=0.745) |
|  | Delta LF Power | 0.26 (p=0.623) | -0.06 (p=0.913) | -0.14 (p=0.787) | -0.20 (p=0.704) | -0.23 (p=0.658) |
|  | Delta HF Power | 0.37 (p=0.468) | 0.23 (p=0.658) | 0.49 (p=0.329) | 0.37 (p=0.468) | -0.32 (p=0.538) |
|  | Delta LF/HF Ratio | -0.26 (p=0.623) | -0.64 (p=0.173) | -0.60 (p=0.208) | -0.60 (p=0.208) | 0.20 (p=0.700) |
| * Statistically significant (p < 0.05). Highlighted in Green. | | | | | | |
| † Approaching statistical significance (p < 0.10). Highlighted in Yellow. | | | | | | |
| Data presented as: Spearman's rho (p-value). | | | | | | |

**
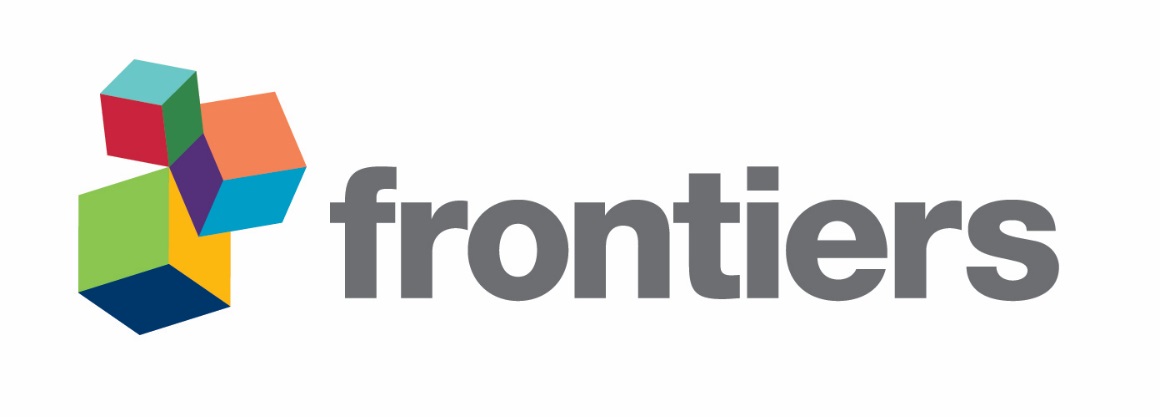
**
